# Peptide-Functionalized Silicon-Photonic E‑Nose for Monitoring Oxidation in Extra Virgin Olive Oil

**DOI:** 10.1021/acsmeasuresciau.6c00021

**Published:** 2026-03-24

**Authors:** Hamed Karami, Antonio Pardo, Luis Fernández, Kaushal Rawal, Santiago Marco

**Affiliations:** † Department of Signal and Information Processing for Sensing Systems, 284118 Institute for Bioengineering of Catalonia (IBEC), The Barcelona Institute of Science and Technology, Baldiri Reixac 10-12, 08028 Barcelona, Spain; ‡ Department of Electronics and Biomedical Engineering, Universitat de Barcelona, Martí i Franqués 1, 08028 Barcelona, Spain

**Keywords:** fraud detection, time-resolved headspace analysis, optoelectronic nose (OE-nose), feature extraction, nondestructive quality control, EVOO

## Abstract

Oxidation is a major factor affecting the quality and shelf life of Extra Virgin Olive Oil (EVOO), leading to chemical degradation and loss of freshness. This study investigates the assessment of EVOO freshness using a peptide-based optoelectronic nose (OE-nose) system combined with signal processing and machine learning techniques. Volatile organic compound (VOC) profiles from fresh and oxidized EVOO samples were acquired using a multigas sensor array implemented on the Aryballe NeOse Advance platform. The oxidation status of the samples was validated using reference chemical quality analyses. Sensor signals were subjected to baseline correction and normalization, without the application of digital smoothing. Full-sequence analysis was employed to exploit desorption-phase kinetics as a volatility-driven, implicit preseparation mechanism, enabling robust discrimination without chromatographic steps. Exploratory and supervised models were evaluated, including principal component analysis (PCA), partial least-squares discriminant analysis (PLS-DA), and support vector machines (SVM). The SVM model achieved a classification accuracy of 100%, while PLS-DA reached 95.8% accuracy under strict validation conditions. Compared to conventional analytical methods, the proposed approach offers a rapid, nondestructive, and cost-effective solution for on-site EVOO freshness evaluation. To the authors’ knowledge, this work represents the first application of a peptide-based optoelectronic nose for assessing EVOO oxidation, highlighting its potential advantages over conventional MOX- and polymer-based electronic nose systems reported in previous studies.

## Graphical abstract

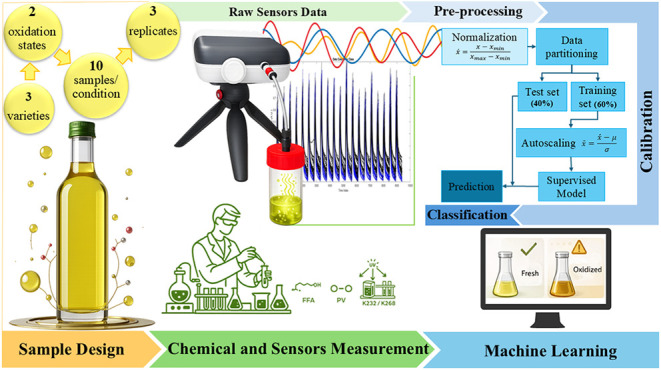

## 1 Introduction

Olive oil, often referred to as the “liquid gold” of the Mediterranean, is treasured not only for its rich flavor but also for its notable health benefits. These benefits largely stem from its high content of monounsaturated fats, vitamin E, and natural antioxidants.^1^ It is rich in oleic acid, a heart-healthy monounsaturated fat known to help reduce the risk of cardiovascular disease, stroke, and overall mortality.^2,3^ Among the different types of olive oil, Extra Virgin Olive Oil (EVOO) holds the highest quality status.^4^ This premium grade is defined by strict requirements, including a low acidity level (below 0.8%), extraction through mechanical means only, and the absence of any sensory flaws.^5^

One of the main factors influencing the quality, safety, and value of EVOO is oxidation. This natural chemical reaction occurs when oxygen interacts with the oil’s unsaturated fatty acids, leading to the formation of various compounds, both volatile and nonvolatile, that negatively affect its flavor, aroma, color, and nutritional content.^6–8^ Detecting oxidation early is vital because once oils begin to oxidize, they lose their fresh, desirable characteristics and the beneficial antioxidants that contribute to health. Moreover, oxidized oils develop unpleasant rancid odors and off-flavors due to the accumulation of volatile secondary oxidation products such as aldehydes, ketones, and short-chain acids. These compounds not only deteriorate the sensory quality and consumer acceptance of the product but may also exert adverse health effects when ingested, including cytotoxic, genotoxic, and pro-inflammatory activities.^9,10^ Consequently, detecting and monitoring lipid oxidation throughout the production, storage, and distribution stages is crucial for ensuring product quality, nutritional integrity, and consumer safety. Beyond quality and safety, oxidation is also relevant to authenticity. Several investigations and regulatory/industry reports link abnormal oxidation/processing markers (e.g., elevated pyropheophytins (PPP), altered 1,2-/1,3-diacylglycerol (DAG) ratios, and specific volatile aldehydes) to fraudulent practices such as selling aged or thermally treated (soft-deodorized) oils as EVOO or blending with refined oils under premium labels. In such cases, oxidation-related signatures act as red flags for misrepresentation and blending, underscoring the need for rapid, nondestructive, and field-deployable monitoring along the supply chain.^11–15^ Several studies have reported successful discrimination of rancidity/oxidation in virgin/extra-virgin olive oil using e-noses, often achieving 80–95% accuracy depending on sensors and protocol; they highlight feasibility but also issues such as drift and cross-sensitivity that limit standardization.^16–18^

Traditionally, evaluating the extent of oxidation in extra virgin olive oil (EVOO) has relied on various chemical and instrumental techniques, as well as sensor-based olfactory platforms (electronic noses) for rapid screening. Trained sensory panels (olfactometry) provide perception-based ground truth but can be variable, resource-intensive, and impractical for rapid, routine checks.^19^ Headspace GC–MS (often SPME-GC–MS) is commonly used to identify key rancidity-related volatiles; GC with FID may be used for targeted quantification, but compound identification typically requires MS.^3,20^ Routine bulk-oxidation indices include the peroxide value (PV; primary products) and p-anisidine value (AV; secondary aldehydes).^21^ Ultraviolet spectrophotometric indices *K*_232_ (conjugated dienes) and *K*_270_ (conjugated trienes) further indicate oxidation progress.^8^ However, these conventional approaches (especially laboratory chemical analyses) are often laborious, require expensive equipment, consume samples, and need specialized laboratory settings,^11^ whereas sensor platforms offer fast, nondestructive fingerprints suitable for routine quality control.

While chemical indices (PV, AV, *K*_232_/*K*_270_) are essential, the legal classification of virgin olive oils in the European Union requires organoleptic (panel) testing, implemented per the IOC method; other jurisdictions may vary in their regulatory adoption.^11,22,23^ In the European Union, the legal classification of virgin olive oils requires organoleptic (panel) testing as laid down in Reg. (EEC) No 2568/91 (Annex XII) and implemented according to the IOC method COI/T.20/Doc. No. 15; this framework is widely described in recent reviews.^11,24^ However, panel testing is demanding and can be affected by assessor subjectivity, training differences, fatigue, and limited throughput, which together challenge reproducibility and routine high-volume screening.^25^

In response to these challenges, there is growing interest in alternative techniques that offer rapid, reliable, and nondestructive analysis of EVOO quality.^26^ Electronic nose (E-nose) technology, which emulates the human sense of smell using sensor arrays and pattern recognition algorithms to detect volatile organic compounds, has emerged as a promising tool.^27,28^ E-nose have previously been applied to assess EVOO oxidation and rancidity, demonstrating good capability in differentiating fresh from oxidized oils.^29–31^

Early studies by Aparicio, Rocha, Delgadillo and Morales^16^ used a 32-sensor conducting-polymer electronic nose with headspace sampling to detect the rancid defect in virgin olive oil; multivariate analysis (e.g., PCA) and pattern-recognition classifiers were trained on mixtures spanning rancidity levels, with sensor selection guided by volatile/panel information (standard baseline/normalization were applied). Subsequent work by Cosio, Ballabio, Benedetti and Gigliotti^32^ combined an electronic nose with an electronic tongue to study storage effects in EVOO, using headspace e-nose data and LDA classification, and showed that e-nose responses alone could reproduce the storage-condition separation (routine centering/scaling prior to LDA). Savarese, Caporaso, Parisini, Paduano, de Marco and Sacchi^33^ Savarese and Caporaso^23^ applied a 10-sensor MOS e-nose with headspace measurements to monitor rancidity and shelf life over time, benchmarking against HS-SPME/GC–MS; data exploration/classification relied on PCA (no digital smoothing beyond typical baseline/scaling). Similarly, Xu, Yu, Liu and Zhang^34^ used a MOS-based e-nose with headspace sampling to qualitatively assess oxidation, employing CA, PCA, and LDA classifiers, with PV/AV as chemical references (standard preprocessing prior to ML). Finally, Santonico, Grasso, Genova, Zompanti, Parente and Pennazza^35^ advanced to a multisensor platform (BIONOTE) fusing gas-phase QMB e-nose data (static headspace) with liquid-phase e-tongue signals; PCA/PLS-DA enabled adulteration detection at low blending levels (baseline correction/centering and cross-validation reported). Most recently, Mariotti, Núñez-Carmona, Genzardi, Pandolfi, Sberveglieri and Mousavi^36^ applied a MOX-based ‘Small Sensor System’ (S3) comprising three chemiresistors (SnO_2_ and Au-doped SnO_2_ operated at 300–400 °C) with autosampler-driven static headspace sampling; sensor features were extracted as ΔR/R_0_ and analyzed by PCA, yielding clear discrimination of ten Umbrian EVOOs and, in some cases, sharper separations than HS-SPME/GC–MS and the sensory panel.

Modesti, Taglieri, Bianchi, Tonacci, Sansone, Bellincontro, Venturi and Sanmartin^37^ showed that hybrid schemes combining instrumental e-noses with human olfactory assessment are promising yet still hampered by calibration and standardization needs; in parallel, the fraud/adulteration outlook by Casadei, Valli, Panni, Donarski, Farrús Gubern, Lucci, Conte, Lacoste, Maquet, Brereton, Bendini and Gallina Toschi,^38^ and the EVOO authentication perspective by Valli, Bendini, Berardinelli, Ragni, Riccò, Grossi and Gallina Toschi^39^ highlight the demand for robust, portable, and intelligent tools that balance speed, cost, and analytical accuracy. Against this backdrop, classical e-noses suffer from long-term drift, cross-sensitivity, and environment-dependent reproducibility, which the community addresses via signal preprocessing (baseline correction, scaling, temporal alignment);^40^ calibration transfer across instruments/conditions (DS, PDS, OSC, GLSW);^41^ and multivariate drift compensation such as CPCA;^42^ alongside newer adaptive/online and domain-adaptation approaches, including deep models.^43^ In contrast, optical sensing platforms functionalized with selective peptide receptors have recently emerged as a promising alternative. By exploiting specific peptide–VOC interactions and photonic transduction mechanisms, these systems can provide enhanced molecular selectivity and improved signal stability compared with conventional MOS or polymeric sensor arrays. Such peptide-based sensing strategies have been increasingly explored for volatile detection in complex chemical environments and offer a potential route toward more robust and reproducible odor sensing systems.^44^ Earlier systems also showed limited sensitivity to subtle changes and often relied on overly simple analyses, restricting uptake in standardized QC.^45^ In response, a peptide-based optoelectronic nose, offering higher selectivity and stability, coupled with modern data analytics, provides a coherent path toward more robust and scalable EVOO oxidation detection.

Peptide-functionalized optoelectronic noses now emerge as a potential solution. These systems offer molecular-level selectivity via engineered peptides and benefit from optical sensing principles. When combined with machine learning classifiers such as Support Vector Machine (SVM) and Partial Least Squares Discriminant Analysis (PLS-DA), they promise exceptional performance in complex, high-dimensional VOC environments. The present research aims to address this gap by employing a state-of-the-art peptide-based optoelectronic nose in combination with signal preprocessing and machine learning techniques, including PCA, PLS-DA, and SVM, to extract rich, meaningful features from sensor data. This method enables a more precise, reliable, and scalable detection of EVOO oxidation, offering significant advantages for producers, regulators, and consumers alike in maintaining product authenticity and safety. This is the first application of a peptide-based optoelectronic nose for EVOO oxidation monitoring, leveraging high molecular selectivity and robust optical sensing for precise volatile profiling.

## 2 Materials and Methods

### 2.1 Samples Preparation

Authentic and adulterated extra virgin olive oil (EVOO) samples were collected to assess oxidation status. The samples comprised different EVOO cultivars, including *Arbequina*, *Picual*, and *Hojiblanca*, which are common commercial varieties in Spain with distinct volatile profiles. A total of 60 independent bottles were analyzed (3 olive oil varieties × 2 oxidation states × 10 samples per variety), with each sample measured in triplicate, resulting in 180 acquisitions. The oxidized samples corresponded to EVOO bottles originally purchased in 2020 and stored in dark conditions at room temperature (approximately 21 ± 1 °C) until the time of analysis, while the fresh samples corresponded to recently produced EVOOs with a production date of 2025. All measurements were conducted under controlled laboratory conditions (21 ± 1 °C, 40% relative humidity). Prior to analysis, oils were stored sealed and protected from light at room temperature to preserve their chemical integrity. All measurements were conducted within 1 week after sample collection. Consequently, the maximum storage duration prior to analysis did not exceed 7 days, thereby minimizing the possibility of additional uncontrolled oxidation during storage. In this study, the term “oxidized samples” refers to oils that exhibited clear signs of oxidative degradation as confirmed by the reference chemical quality parameters (PV, *K*_232_, and *K*_268_) exceeding the IOC limits. The oxidation state was therefore defined based on chemical analysis rather than artificially accelerated oxidation procedures.

For measurements, 20 mL of each sample was transferred into 60 mL airtight glass vials, providing sufficient headspace for volatile compound accumulation. The vials were sealed and equilibrated at room temperature for 30 min to allow volatile organic compounds (VOCs) to reach a stable headspace concentration. The acquisition order was randomized using a computer-generated sequence to minimize systematic bias. This protocol ensured reproducible and representative headspace compositions for reliable sensor-based measurements.

For chemical quality assessment, a separate aliquot of 60 mL from each oil sample was used to perform the reference analyses, including free fatty acids (FFA), peroxide value (PV), and specific extinction coefficients (*K*_232_ and *K*_268_), according to the official International Olive Council (IOC) methods. These chemical analyses were carried out independently from the sensor measurements to avoid cross-contamination and to ensure accurate evaluation of the oxidation status of each sample.^46,47^

### 2.2 Reference Chemical Analyses

#### 2.2.1 Determination of Free Fatty Acids (FFA)

The free fatty acid content of the oil samples was determined according to the official method of the International Olive Council (IOC), COI/T.20/Doc. No 34.^48^ Briefly, a known amount of oil sample was dissolved in an appropriate solvent mixture and titrated with a standardized potassium hydroxide (KOH) solution. Phenolphthalein was used as the indicator to determine the titration end point. The results were expressed as percentage of oleic acid. According to IOC standards, oils with FFA values higher than 0.8% (as oleic acid) indicate quality deterioration and possible hydrolytic degradation.

#### 2.2.2 Determination of Peroxide Value (PV)

The peroxide value was measured following the IOC official method COI/T.20/Doc. No 35.^49^ In this procedure, the oil sample was dissolved in a mixture of acetic acid and chloroform, followed by reaction with potassium iodide. The liberated iodine was titrated with a standardized sodium thiosulfate solution. The peroxide value was expressed as milliequivalents of active oxygen per kilogram of oil (meq O_2_/kg oil). According to IOC standards, a peroxide value exceeding 20 mequiv O_2_/kg oil is considered an indicator of advanced primary oxidation.

#### 2.2.3 Determination of Specific Extinction Coefficients (*K*_232_ and *K*_268_)

Specific extinction coefficients at 232 nm (*K*_232_) and 268 nm (*K*_268_) were determined according to the IOC method COI/T.20/Doc. No 19.^50^ Oil samples were diluted in isooctane, and absorbance was measured using a UV–Vis spectrophotometer at the specified wavelengths. The coefficients were calculated following the IOC formula and reported as indicators of primary and secondary oxidation products. According to IOC standards: *K*_232_ values higher than 2.50 indicate increased formation of conjugated dienes (primary oxidation), *K*_268_ values higher than 0.22 indicate the presence of secondary oxidation products.

### 2.3 Instrumentation and Measurement Conditions

2.3.1 Measurements were performed with NeOse Advance (Aryballe, Grenoble, France), which integrates a Core Sensor Module (CSM) based on silicon photonics and an internal fluidic system (vacuum pump, valves) with two inlets (baseline/sample). Factory flow is 60 mL/min (adjustable). Control and acquisition were handled via Aryballe Suite. A standard warm-up (one cycle, then ∼30 min to thermal equilibrium) was applied; samples were kept at or below instrument temperature to avoid condensation.^44^

The CSM comprises an array of Mach–Zehnder interferometers (MZIs) whose phase shifts with changes in the effective refractive index near the waveguides. Peptide receptors functionalized on the sensing paths interact with VOCs, altering the local index and yielding a time-resolved interferometric response. Owing to their diverse, cross-reactive affinities, the multichannel pattern constitutes an odor fingerprint subsequently modeled by multivariate methods (e.g., PCA, PLS-DA, SVM). The baseline line (PTFE-filtered) supplies a clean reference for drift control; the sample line draws vial headspace via PEEK tubing (needle through septum for pressure equalization). Each measurement followed a fixed baseline,exposure,desorption cycle: baseline: 10 s, analyte: 30 s at 15 mL/min, desorption/purge: 120 s. All channels were recorded simultaneously throughout the cycle.

### 2.4 Data Preparation

Before constructing the feature space, the sampling procedure was carefully designed to include three distinct phases: (i) baseline, where the sensors were exposed to clean carrier gas to stabilize the signal; (ii) analyte exposure, during which the volatile compounds from the sample interacted with the sensing surface, producing a characteristic increase in signal amplitude; and (iii) desorption, where the carrier gas was reintroduced to purge the sensors and allow recovery toward the baseline. A representative sensor response is depicted in Figure 1, showing the temporal evolution of the signal amplitude across these three phases. To retain the full dynamics of the sensing process, the entire time-resolved responses from all 20 sensors, including baseline, analyte exposure, and desorption phases, were utilized for each measurement.

**Figure 1:**
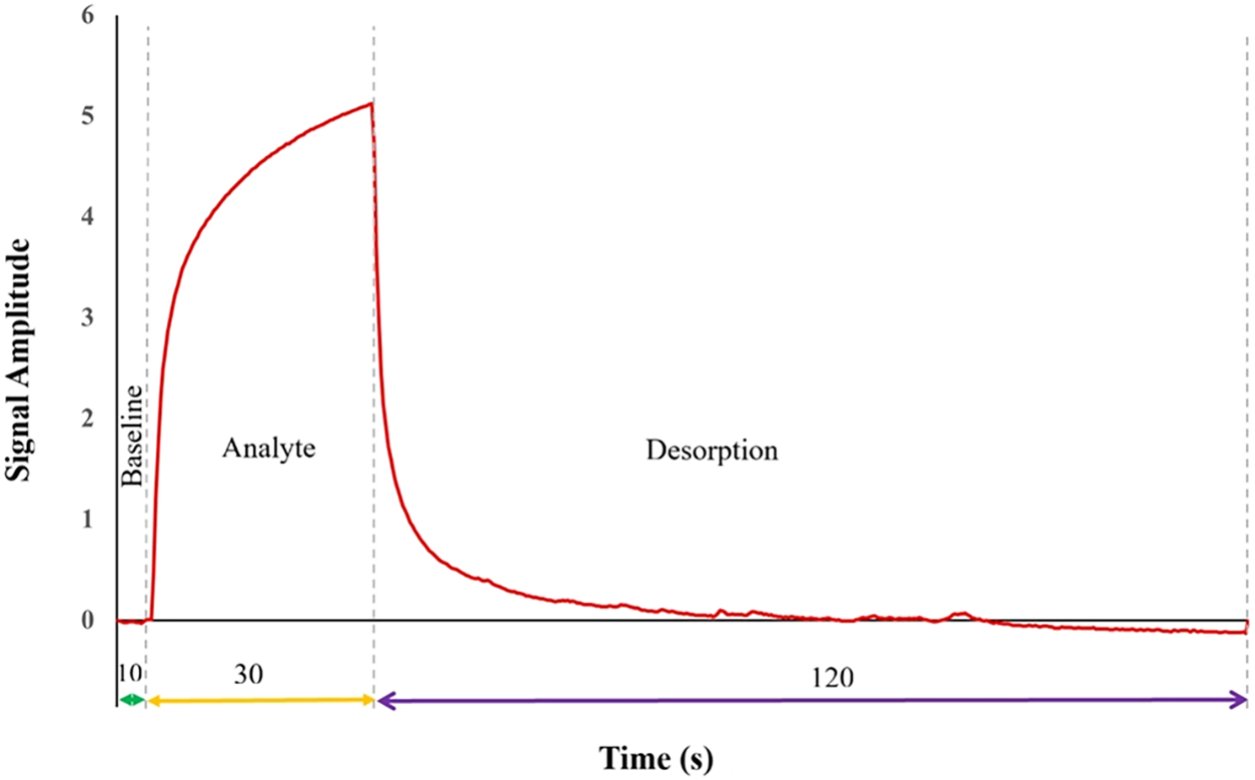
Typical time-resolved sensor response illustrating the three phases of the sampling cycle.

Before preprocessing, the raw time-resolved outputs from all sensors were concatenated sequentially across time, forming a single high-dimensional feature vector that captured both temporal and sensor-specific variations. This procedure resulted in a total of 9380 features per sample, providing a comprehensive representation of the sensing event and enabling more effective pattern recognition. However, the raw signals often exhibited baseline drift, noise, and intersensor variability, which can negatively impact classification performance. To enhance data quality and ensure consistency, several signal preprocessing steps were applied. Digital smoothing was intentionally not applied in order to preserve the original time-resolved dynamics of the sensor responses, particularly during the adsorption and desorption phases. Preliminary tests indicated that smoothing did not improve class separation and could attenuate subtle kinetic features that contribute to the discrimination between fresh and oxidized samples. First, baseline correction was performed by subtracting the initial sensor response before analyte exposure, minimizing sensor offset. Subsequently, min–max normalization was used to rescale the data to a uniform range, improving comparability among sensors with different dynamic ranges, where each feature was rescaled to the range [0,1] according to

1 $$\mathrm{x́}=\frac{x-x_{\min}}{x_{\max}-x_{\min}}$$

where *x* = the original raw sensor value, *x*_min_ = the minimum value of the feature across the data set, *x*_max_ = the maximum value of the feature across the data set, and *x*′ = the normalized value of the feature in the range [0,1], This transformation removes baseline offsets and ensures comparability among sensors with different dynamic ranges. This step harmonizes the amplitude scale of all sensor responses and prevents sensors with larger raw signals from dominating the feature space. After normalization, autoscaling (mean-centering followed by division by the standard deviation) was applied to standardize feature variance across sensors and time points. The combination of min–max normalization and autoscaling is particularly suitable for high-dimensional time-series sensor data, as it ensures both comparable dynamic ranges and balanced statistical contribution of each feature during multivariate modeling.

2 $$ẍ=\frac{\mathrm{x́}-\mu }{\sigma }$$

where *x*″ is autoscaled value, μ is the mean of the feature and σ is its standard deviation. Autoscaling standardizes the features, ensuring equal contribution of each sensor to the multivariate analysis regardless of their original variance.

### 2.5 Multivariate and Machine Learning Analysis

Following signal preprocessing, the complete response curve from each sensor, including the baseline, analyte exposure, and desorption phases, was used for analysis. Rather than isolating individual time segments, the full signal shape was retained to capture the complete dynamic behavior of each sensor. To analyze the extracted features, several multivariate statistical and machine learning methods were applied using the PLS Toolbox in MATLAB (eigenvector Research Inc., USA).

Principal Component Analysis (PCA) was first used as an unsupervised exploratory technique to reduce data dimensionality and uncover underlying patterns in the data set.^51,52^ By projecting the high-dimensional feature space onto a smaller number of principal components that capture the greatest variance, PCA enabled visualization of sample distributions and potential clustering, helping to identify separability between fresh and oxidized EVOO samples.

For classification purposes two supervised classification methods were tested. To evaluate classification models, confusion matrices were generated as tabular summaries comparing predicted sample labels against true classes.^53^ This allowed calculation of true positives, false positives, true negatives, and false negatives, providing a detailed view of model performance on each category.^54^ Quantitative metrics derived from the confusion matrix, including accuracy, sensitivity (recall), specificity, precision, and F1-score, were computed to assess classifier effectiveness comprehensively.^55^ To validate the models and prevent overfitting, we applied a Venetian-blind cross-validation strategy with 10 folds and a segment thickness of one, as implemented in the PLS Toolbox. This method ensures that samples are systematically left out across the measurement sequence, providing a balanced and unbiased assessment of model performance. Regarding replicates, we considered the three replicate measurements of each oil sample as belonging to the same class and ensured that all replicates from a given sample were kept within the same fold during cross-validation. This approach avoids information overfitting between training and validation sets, thereby ensuring that the reported classification results truly reflect model generalizability to unseen samples. Model performance was evaluated using standard diagnostic criteria, including accuracy, precision, recall (sensitivity), specificity, F1-score, and AUC, following previously established definitions.^56^

Partial Least Squares Discriminant Analysis (PLS-DA) to address the challenges of multicollinearity and sensor noise, PLS-DA was applied as a supervised modeling approach.^27,57^ It identifies latent variables that explain the covariance between predictors and class labels, providing a compact representation of the data suitable for supervised classification in high-dimensional and noisy feature spaces Support Vector Machine (SVM) with a linear kernel, was implemented to enhance classification performance further.^28,58^ SVM constructs an optimal hyperplane in a transformed feature space to maximize the margin between classes, making it well-suited for handling complex, nonlinear patterns often encountered in VOC-based sensor data. The overall analytical workflow, including data preprocessing, data set partitioning, model calibration, and validation, is illustrated in Figure 2. For model development, the data set was divided into a training set (38 samples, 60%) and an independent test set (22 samples, 40%) following a user-defined split to ensure balanced representation of both classes. To prevent bias and overfitting, all replicates from the same oil sample were assigned to the same subset (either training or test). The training set was used for model calibration and internal user-defined cross-validation, while the independent test set was reserved exclusively for external validation and performance assessment. The hyperparameters of the classifiers were optimized by minimizing the balanced classification error rate, calculated as the average of false positive and false negative rates.

**Figure 2:**
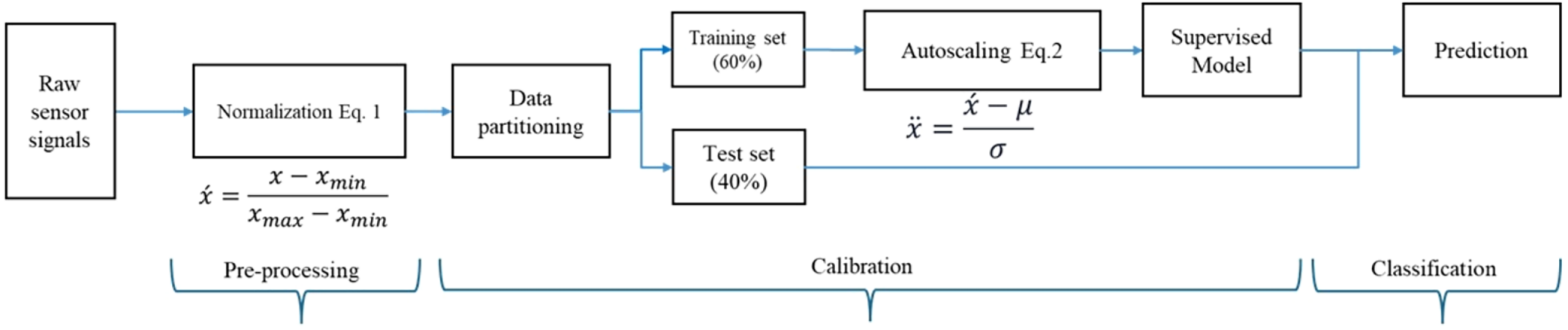
Workflow of data preprocessing, partitioning, model calibration, and validation in the PLS-DA and SVMDA classification framework.

To account for uncertainty arising from the limited sample size, confidence intervals (CIs) were computed for all reported classification metrics. For discrete metrics derived from the confusion matrix (accuracy, precision, recall/sensitivity, and specificity), 95% confidence intervals were estimated using the exact binomial (Clopper–Pearson) method. The F1-score, which is not a simple binomial proportion, was evaluated using bootstrap resampling (*B* = 2000). For ROC analysis, the area under the curve (AUC) and its confidence interval were estimated using an exact binomial approach based on all pairwise comparisons between positive and negative samples, providing a conservative assessment of ranking performance under finite-sample conditions.

## 3 Results and Discussion

### 3.1 Chemical Quality Parameters and Oxidation Status

The chemical quality parameters of the analyzed oxidized olive oil samples are presented in Table1 (Supporting Information). The results clearly indicate that all analyzed samples corresponding to *Arbequina*, *Hojiblanca*, and *Picual* cultivars exhibited advanced oxidation when compared with the fresh extra virgin olive oils (EVOO).

The free fatty acid (FFA) content of the oxidized samples ranged from 0.43 to 0.45%, remaining below the IOC regulatory limit of 0.8% for EVOO. This suggests that hydrolytic degradation was not the dominant deterioration mechanism and that the observed quality loss was primarily associated with oxidative processes rather than triglyceride hydrolysis.

In contrast, the peroxide values (PV) of samples were substantially higher than the IOC maximum limit of 20 mequiv O_2_/kg oil, with measured values of 25.8 ± 0.4, 22.6 ± 0.1, and 21.6 ± 0.1 mequiv O_2_/kg for *Arbequina*, *Hojiblanca*, and *Picual* oils, respectively. These elevated PVs indicate intense primary oxidation and accumulation of hydroperoxides.

Similarly, the specific extinction coefficients *K*_232_ and *K*_268_ showed markedly elevated values compared with those reported for fresh EVOO. The *K*_232_ values (3.6–4.7) were well above the IOC limit of 2.50, reflecting a high concentration of conjugated dienes formed during primary oxidation. Moreover, K268 values (0.35–0.38) exceeded the regulatory threshold of 0.22, indicating the presence of secondary oxidation products such as aldehydes and ketones.

When compared with values for fresh *Arbequina*, *Picual*, and *Hojiblanca* oils, which typically exhibit PV < 12, *K*_232_ < 2.2, and *K*_268_ < 0.20, the analyzed samples demonstrate a clear deviation from the chemical profile of fresh EVOO. These results confirm that the samples can be reliably classified as oxidized oils, providing a robust chemical ground truth for subsequent sensor-based analyses.

### 3.2 Signal Representation and Feature Construction

Figure 3 shows how each sensor reacted during the desorption step, with fresh oils colored blue and spoiled ones colored yellow. The horizontal axis represents the number of features for 20 sensors, totaling 9380 features, which corresponds to a time span of 160 s per measurement cycle. Since the signals of all 20 sensors were concatenated sequentially, the *x*-axis simultaneously reflects both the temporal evolution within each cycle and the ordering of the sensors. The vertical axis indicates signal strength in arbitrary units (A.U.). Every tip in the curve lines up with a complete measurement sweep, and the desorption phase is clear as the line drops sharply while the volatile compounds leave the chamber. Fresh samples fade fast and evenly, while oxidized oils cling to a higher signal for longer, proving they carry heavier, tangled oxidation leftovers. As oils undergo oxidation, they form various compounds like hydroperoxides, aldehydes, ketones, free fatty acids, and even larger molecules such as polymers. Oxidation generates heavier oligomers/dimers that increase bulk viscosity, while simultaneously altering the volatile fingerprint (e.g., aldehydes and ketones).^59^ These changes do not reflect a direct measurement of bulk physical properties such as viscosity. Rather, the sensor system responds to alterations in the volatile chemical profile generated during lipid oxidation, including aldehydes, ketones, and other secondary oxidation products that modify the headspace composition of the oil samples. The oxidation-induced shift in the overall volatile pattern produces reproducible signal changes. These differences enable reliable classification of fresh versus oxidized oils using pattern-recognition methods. Employing multiple absorption and desorption cycles provides replicate time windows of the same headspace event. Aggregating information across cycles improves the signal-to-noise ratio and buffers minor within-run baseline/flow fluctuations (e.g., slight offsets between consecutive windows). Consequently, the models exploit spatiotemporal structure, amplitude, slope, area, and decay, not merely a single instantaneous peak value. Peak-only summaries primarily capture instantaneous concentration/affinity and are more sensitive to minor baseline or flow variations, while being blind to kinetic separability. When these time-resolved cues are combined across 20 sensors and cycles (9,380 features over 160 s), they yield stable, discriminative patterns that explain the strong test-set performance reported here. Although a formal benchmark against single-cycle or peak-only features was not the aim of this study, the observed results support the choice of analyzing the complete sequence and motivate future ablations to quantify the contribution of each phase and feature family. The clear features that distinguish the two oil groups show that chemometric tools such as PCA or PLS-DA can successfully sort them and assess their actual freshness.

**Figure 3:**
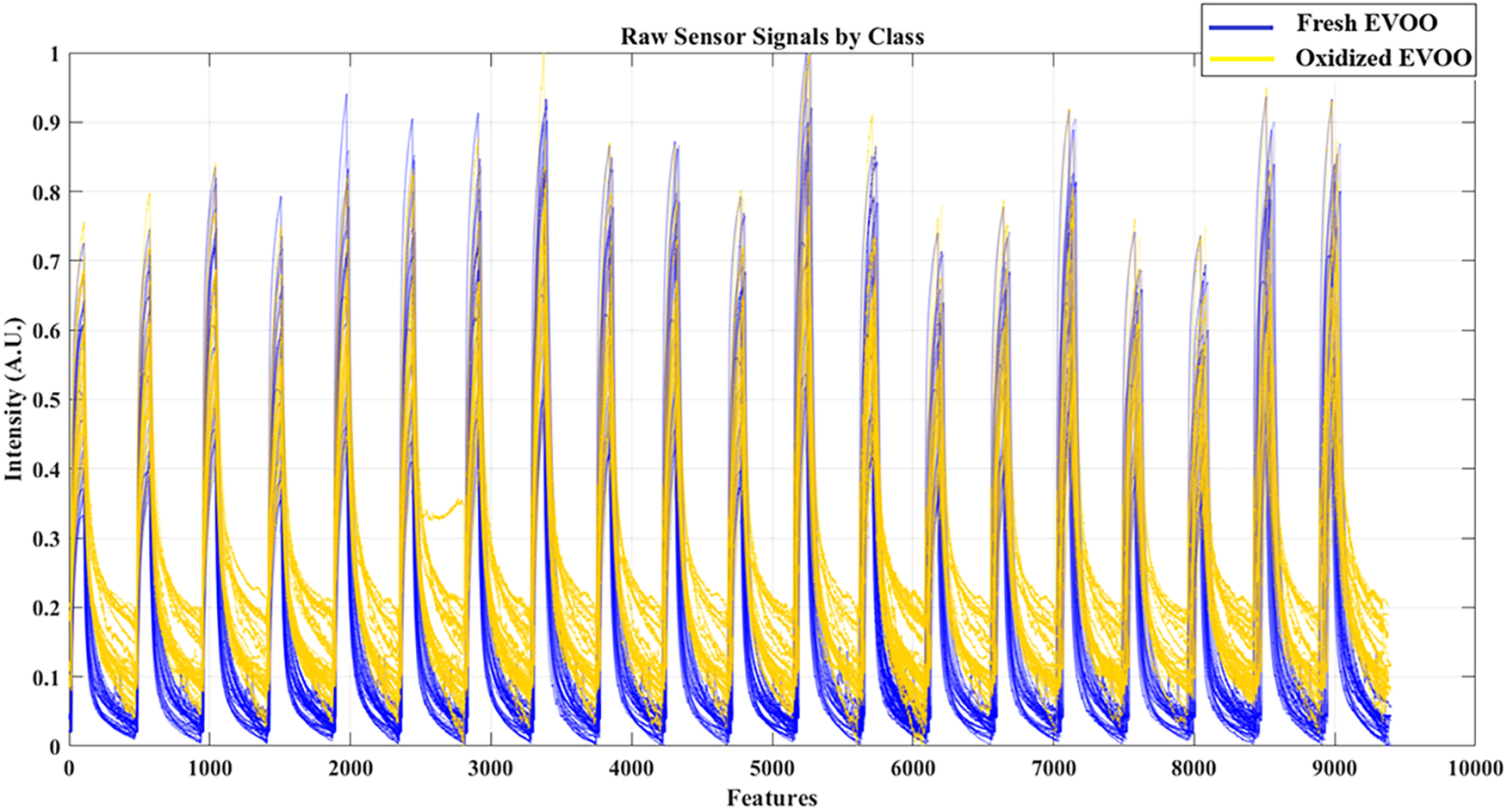
Time-series responses for 20 sensors across baseline, exposure, desorption (170 s total; 9380 concatenated features).

### 3.3 Principal Component Analysis (PCA) Results

In this study, we used PCA to analyze data obtained from the electronic nose, aiming to identify hidden patterns and reduce data volume for easier processing and interpretation. The main reason for this was to decrease data complexity, as feature vector had 9380 features. As an unsupervised method, PCA identifies directions in the data with the greatest variation and then transforms the original variables into a smaller set while preserving the essential information.

We performed PCA using the singular value decomposition (SVD) method. The results showed (Figure 4a) that the first principal component (PC1) explained 69% of the total variance, while the second principal component (PC2) explained an additional 15%, resulting in a total explained variance of 85%. This indicates that most of the important information from the original data was captured by only the first two components. As shown in Figure 4a, the separation between fresh and oxidized oil samples is clearly visible. Fresh oils (blue) cluster on the left, while oxidized oils (yellow) spread out on the right. This distinct grouping demonstrates that the electronic nose effectively distinguishes between the two types of oils. The score plot reveals a clear grouping of the samples according to their oxidation state, demonstrating that the electronic nose effectively distinguishes between the two oil conditions. This result confirms the discriminative capability of the sensor system and highlights PCA as a useful exploratory tool for visualizing class separation in high-dimensional sensor data. The dominant separation observed along PC1 is mainly associated with oxidation-related changes in the volatile profile, which are linked to the formation of secondary oxidation products such as aldehydes and ketones. In contrast, the variation captured by PC2 mainly reflects residual variability within the data set rather than a clear discrimination factor, as the spread of the sample clouds largely overlaps across oil types. This observation is consistent with the subsequent PLS-DA results, where a single latent variable was sufficient to achieve class discrimination.This result not only confirms the discriminative capacity of the sensor system but also highlights PCA as a valuable tool for visualizing class separation in high-dimensional data.

**Figure 4:**
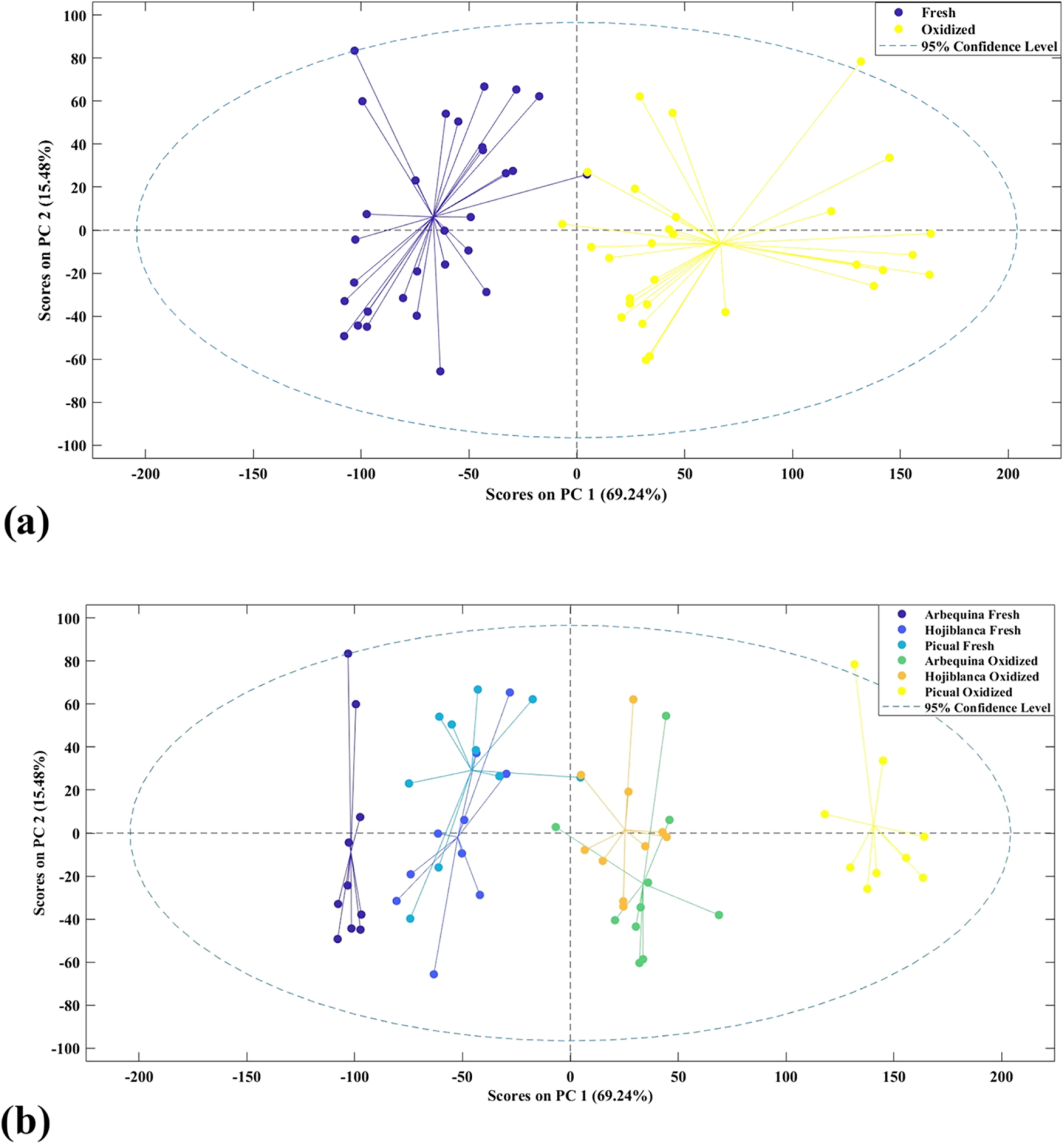
PCA score plots of e-nose data (SVD-based PCA). (a) Two-class view (Fresh vs Oxidized), and (b) Six-class view by cultivar × condition (*Arbequina*, *Hojiblanca*, *Picual*; fresh/oxidized). The first two principal components explain 85% of the total variance (PC1:69%, PC2:16%).

Moreover, as shown in Figure 4b, when labeling by cultivar (e.g., *Arbequina*, *Hojiblanca*, *Picual*) and condition (fresh/oxidized), subclusters emerge within each global class. Fresh oils from different cultivars remain on the negative side of PC1 but are separated along PC2, reflecting cultivar-specific baselines in volatile profiles. Oxidized oils occupy the positive side of PC1 yet form distinct varietal subclusters, again dispersed primarily along PC2. Thus, the broader left–right split (PC1) is driven by oxidation chemistry, whereas the vertical spread (PC2) captures varietal effects. Notably, oxidized groups exhibit slightly greater radial dispersion than fresh groups, consistent with cultivar-dependent oxidation kinetics and heterogeneous formation of secondary oxidation products. Overall, the PCA confirms that (i) oxidation is the dominant source of variance, cleanly separating samples along PC1, and (ii) residual structure within each class is explained by variety, which accounts for the observed within-class scatter. This unsupervised evidence aligns with the supervised results (PLS-DA and SVM), reinforcing that the e-nose signal encodes both oxidation status and cultivar signatures.

In the loading plot Figure 5, the adsorption, desorption cycles are visible for all 20 sensors; the peaks correspond to the adsorption phase and the troughs to desorption. The peaks are the windows that explain oxidized oils and align with the positive PC1 direction on the right side of the PCA plot; the troughs mainly describe the behavior of fresh oils and are consistent with the negative/left side of PCA. The advantage of this e-nose lies precisely in these adsorption–desorption cycles, which, due to differences in the volatility/molecular mass of oil constituents, create a kind of time-based ‘preseparation’ that enables discrimination. Since these cycles have a similar shape across channels, even a well-chosen single peptide could account for much of the classification power; therefore, given the technology’s cost, reducing the number of sensors could make the system more practical and cost-effective for many applications.

**Figure 5:**
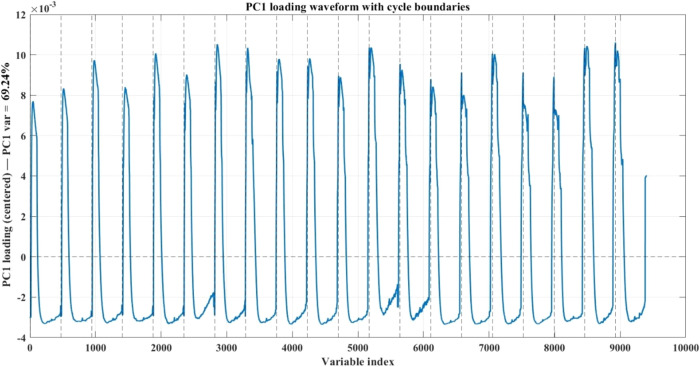
PC1 loading vector (explained variance = 69.24%).

### 3.4 Partial Least Squares Discriminant Analysis (PLS-DA) Results

Figure 6a presents the PLS-DA score plot, illustrating the distribution of two groups of oil samples, fresh and oxidized, based on the first two latent variables, LV1 and LV2. The analysis was performed using the training and test partitions described in the Methods section. In the plot, solid markers represent training samples, while hollow markers correspond to test samples. As shown in Figure 6a, the horizontal axis (LV1) and vertical axis (LV2) represent the first and second latent variables, respectively. The two groups are clearly separated along the LV1 axis, indicating that the PLS-DA model effectively distinguishes between fresh oils (blue, left side) and oxidized oils (yellow, right side). The most significant discriminative power is observed along LV1, where the majority of the group separation occurs. The alignment between training and test samples demonstrates the model’s stability and generalizability. Test samples (hollow markers) fall within their respective group regions, confirming that the model was not overfitted and can be reliably applied to unseen data. Although Figure 6 displays two latent variables (LV1 and LV2) for visualization purposes, the optimal number of latent variables was determined to be one (LV = 1) based on RMSECV minimization (RMSECV = 0.06), indicating that one LV was sufficient to capture the relevant covariance between the sensor data and the class labels. Adding further latent variables did not improve classification performance and increased the risk of overfitting. Accordingly, a parsimonious PLS-DA model with one LV was selected. The model’s predictive performance was further supported by a low prediction error (RMSEP = 0.17) for the training set and RMSEP = 0.0000 for the test set. These results indicate that a single latent variable was sufficient to capture the relevant variance and achieve optimal discrimination between fresh and oxidized oil samples.

**Figure 6:**
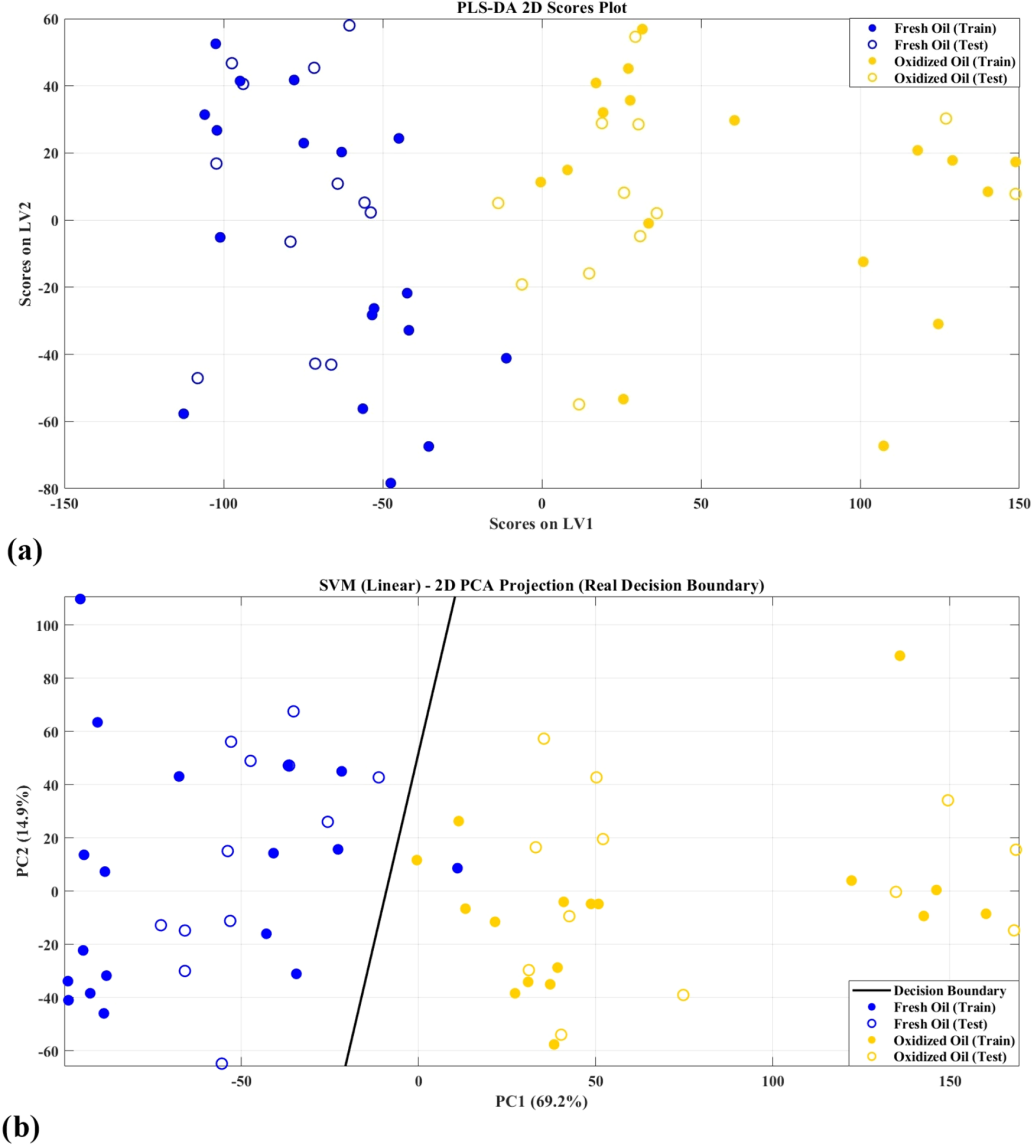
Classification results of the (a) PLS-DA and (b) SVMDA model for showing separation between fresh and oxidized oil samples.

The confusion matrices for both the training and test sets are presented in Table 1. Using one latent variable (LV), the model achieved high discrimination between fresh and oxidized oils. On the training set (*n* = 36), accuracy was 0.97, precision 0.95, recall (sensitivity) 1.00, specificity 0.944, F1-score 0.973, and AUC 1.000; the confusion matrix showed a single misclassification (fresh to oxidized). On the independent test set (*n* = 24; 12 per class), accuracy was 0.96, precision 1.00, recall 0.917, specificity 1.000, F1-score 0.957, and AUC 1.00; the confusion matrix indicated one oxidized sample predicted as fresh (12/12 fresh correctly identified; 11/12 oxidized correctly identified). To quantify uncertainty due to the limited test size, 95% exact binomial confidence intervals were computed for discrete metrics: accuracy 0.96 (95% CI: 0.79–0.99), precision 1.00 (0.71–1.00), recall 0.92 (0.62–0.99), and specificity 1.00 (0.73–1.00). For the ROC analysis, the confidence interval of the AUC was computed using an exact binomial (Clopper–Pearson) approach based on all pairwise comparisons between positive and negative samples. Given that all 144 (12 × 12) comparisons were correctly ranked, the AUC was 1.00 with a 95% confidence interval of approximately 0.98–1.00. Bootstrap resampling (*B* = 2000) yielded an F1-score of 0.957 (95% CI: 0.84–1.00), corroborating excellent generalization with a single borderline error on the test set. Model parsimony and stability were further supported by low cross-validated and external prediction errors (RMSECV = 0.06 at the optimal LV; RMSEP = 0.17 for training predictions and 0.20 on the test set). Collectively, these results indicate a compact, robust classifier with near-perfect discrimination for detecting oil oxidation, well suited for nondestructive EVOO quality assurance and fraud detection.

**Table 1:** Chemical Quality Parameters of Oxidized EVOO Samples^a^

Cultivar	FFA (% oleic acid)	PV (meq O_2_/kg oil)	*K*_232_	*K*_268_
*Arbequina*	0.45 ± 0.01	25.8 ± 0.4	4.7 ± 0.0	0.36 ± 0.0
*Hojiblanca*	0.45 ± 0.01	22.6 ± 0.1	4.3 ± 0.1	0.38 ± 0.0
*Picual*	0.43 ± 0.00	21.6 ± 0.1	3.6 ± 0.1	0.35 ± 0.0

a Results expressed as mean ± standard deviation of duplicates.

### 3.5 Support Vector Machine (SVM) Classification Results

In this study, a classification model named SVMDA (Support Vector Machine Discriminant Analysis) was constructed to discriminate between two groups of oil samples. The model was developed using the C-Support Vector Classification (C-SVC) framework and employed a linear kernel, which proved sufficient to achieve complete separation between the two categories. Preliminary testing with nonlinear kernels (e.g., RBF) did not provide any improvement in classification performance, while increasing model complexity. Therefore, the linear kernel was selected as a simpler and more interpretable solution, consistent with the clear linear separability observed in the PCA results. The optimal regularization parameter was identified as a cost (C) of 0.003. This small value of C imposes a softer margin on the classification boundary, meaning that the model tolerates small errors in order to improve generalization. In practice, this prevents the decision boundary from being overly sensitive to noise or minor fluctuations in the high-dimensional sensor data. The use of a linear kernel, together with a low cost parameter, suggests that the two EVOO categories were linearly separable in the feature space after autoscaling, eliminating the need for more complex nonlinear kernels such as RBF. The final model required only six support vectors, which is a very small fraction of the total number of training samples. This indicates that only a handful of samples were critical in defining the decision boundary, while the majority of data points lay at a safe distance from the separating hyperplane. Such an outcome reflects the strong intrinsic separability of the data set. The decision boundary and classification results are illustrated in Figure 6b, which clearly shows the complete separation of the two groups without any overlap. Importantly, the circled points representing the independent test set fall within the same class clusters as the training data, confirming the model’s excellent generalization ability. Moreover, the elliptical confidence regions surrounding each class show no overlap, further supporting the robustness and reliability of the linear kernel in capturing the intrinsic data structure. This graphical representation confirms that the linear kernel was sufficient to model the structure of the data, providing a simple yet highly effective classification surface.

The confusion matrix (Table 1) indicates perfect separation of classes in test phases: all fresh oil samples were assigned to class 1 and all oxidized samples to class 2, with no misclassifications. Consequently, the true positive rate (TPR) and true negative rate (TNR) were 1.00, whereas the false positive rate (FPR) and false negative rate (FNR) were 0.00. Precision, recall (sensitivity), and F1-score were 1.00 for both classes, and the Matthews Correlation Coefficient (MCC) reached its maximum of 1.000, evidencing unbiased, perfectly concordant predictions. To quantify uncertainty given the limited test size (*n* = 24; 12 per class), 95% confidence intervals were estimated using the exact binomial model: overall accuracy was 1.00 with a 95% CI of 0.86–1.00, while per-class metrics based on 12/12 successes (precision, recall, and specificity) were 1.00 with 95% CIs of 0.73–1.00.

Comparison of classification models (Table 2). Both models showed strong performance on extra virgin olive oil (EVOO) discrimination, with the SVM outperforming PLS-DA on the test set. For training (*n* = 36), PLS-DA with one latent variable achieved 97.2% accuracy (one fresh sample misclassified), whereas the linear SVM reached 100% accuracy with no errors. On the independent test set (*n* = 24; 12 per class), SVM delivered perfect classification across all metrics (accuracy, precision, recall, specificity, F1, AUC = 1.00). In contrast, PLS-DA achieved high, but not perfect, performance: accuracy 0.96, precision 1.00, recall 0.92, specificity 1.00, F1 0.96, and AUC 1.00; the single error corresponded to one oxidized sample predicted as fresh. To reflect uncertainty due to sample size, 95% exact binomial confidence intervals on the PLS-DA test metrics were: accuracy 0.79–0.99, precision 0.71–1.00, recall 0.62–0.99, and specificity 0.73–1.00; bootstrap CIs (*B* = 2000) were 0.98–1.00 for AUC and 0.84–1.00 for F1. Collectively, these results indicate that while both approaches generalize well, SVM achieves flawless discrimination on the available data, and PLS-DA provides a compact, near-perfect alternative with strong robustness.

**Table 2:** Test-set Performance with 95% Cis Extracted for PLS-DA and SVMDA Method for Classification of Two Groups of EVOO

Models	Metric	Point Estimate	CI_Low	CI_High	Notes (*k*/*n*)
PLS-DA	Accuracy	0.96	0.79	0.99	23/24
	Precision	1	0.71	1	11/11 (positive)
	Recall	0.92	0.62	0.99	11/12 (positive)
	Specificity	1	0.73	1	12/12 (negative)
	F1	0.96	0.84	1	bootstrap (B = 2000)
	AUC	1	0.98	1	Clopper–Pearson exact CI (12 × 12 = 144 comparisons)
SVMDA	Accuracy	1	0.86	1	24/24
	Precision	1	0.73	1	12/12 (positive)
	Recall	1	0.73	1	12/12 (positive)
	Specificity	1	0.73	1	12/12 (negative)
	F1	1	1	1	bootstrap (B = 2000)
	AUC	1	0.98	1	Clopper–Pearson exact CI (12 × 12 = 144 comparisons)

The classification performance observed in this study is comparable to or slightly higher than results reported for many MOS-based electronic nose systems applied to olive oil quality assessment, where classification accuracies typically range between 80% and 95% depending on sensor configuration and experimental protocol.^17,36,60^ For example, Poeta, Núñez-Carmona, Sberveglieri, Bernal, Lozano and Sánchez^61^ demonstrated that a MOX-based electronic nose could discriminate between extra virgin olive oil, olive oil, and olive pomace oil using volatile fingerprints. Their system achieved classification accuracies above ∼90%, highlighting the capability of MOS sensor arrays for rapid and nondestructive olive oil authentication. In comparison, the peptide-functionalized optoelectronic platform used in the present work exploits molecularly selective peptide receptors combined with photonic transduction, which may contribute to enhanced sensitivity to oxidation-related volatile signatures. While both approaches demonstrate the feasibility of sensor-based olive oil classification, the present system emphasizes high molecular selectivity and time-resolved signal analysis to capture oxidation-induced changes in the volatile profile. The strong performance obtained here may be attributed to the combined effect of peptide-functionalized sensing elements and the exploitation of full time-resolved response dynamics, which together provide a richer representation of the volatile fingerprint associated with oxidation processes.

## 4 Conclusion

This study demonstrates that a peptide-based optoelectronic e-nose, combined with standard chemometric methods, can reliably detect extra virgin olive oil (EVOO) oxidation from time-resolved headspace signals. Using 60 independent bottles across three cultivars (fresh vs oxidized; triplicate acquisitions; 9,380 time-series features per sample), two supervised classification models were evaluated. On an independent test set (*n* = 24), a linear SVM achieved flawless discrimination on the available data, with no misclassifications observed across all metrics (accuracy, precision, recall, F1-score, and AUC = 1.00). The associated 95% exact binomial confidence intervals (e.g., accuracy 0.86–1.00) appropriately reflect the uncertainty arising from the limited test size. In comparison, PLS-DA delivered near-perfect performance (accuracy 0.96, precision 1.00, recall 0.92, specificity 1.00, and AUC 1.00), with a single oxidized sample misclassified as fresh. For ROC analysis, uncertainty in the AUC estimate was quantified using an exact binomial (Clopper–Pearson) approach based on all pairwise comparisons between positive and negative samples, yielding AUC = 1.00 with a 95% confidence interval of approximately 0.98–1.00. This conservative estimate supports the robustness of class separation while avoiding overinterpretation of perfect ranking under finite sample conditions. A key driver of the strong classification performance lies in the acquisition protocol itself. Repeated absorption–desorption cycles provide an implicit preseparation by volatility, with the desorption phase being particularly informative. By analyzing the full baseline–exposure–desorption sequence rather than a single steady-state peak, the models exploit time-resolved features such as amplitude rise, slope, area under the response curve, and the desorption decay constant (τ). Concatenation of multiple cycles across the 20-sensor array improves signal-to-noise ratio, enhances stability, and yields reproducible spatiotemporal response patterns that enable robust discrimination. Collectively, these findings indicate that compact, well-regularized linear models can extract reliable pattern-level information from peptide-functionalized, optically transduced sensor arrays, enabling fast and nondestructive screening of EVOO freshness. Beyond classification accuracy, the platform’s compact instrumentation and straightforward workflow support potential deployment at the point of need across production, storage, and retail environments. Although the present results are encouraging, the study was conducted on a relatively limited number of samples and under controlled laboratory conditions. Future work will therefore include a larger data set encompassing a broader range of olive oil cultivars and storage histories, as well as validation under real-world environmental conditions. In addition, multisite and multi-instrument studies with calibration transfer will be explored, together with long-term evaluation of sensor stability. These efforts are essential to translate the demonstrated laboratory performance into scalable and standardized tools for routine EVOO quality control and fraud prevention.

## Supplementary Material



## Data Availability

The data are available from the corresponding author on reasonable request.

## Notes

Specifically, it provides a description of the cross-validation strategy and data partitioning used in this study (Text S1); details on latent variable selection and model parsimony in the PLS-DA model (Text S2); and the full confusion matrices for the PLS-DA and SVM models evaluated on the independent test set (Table S1) (PDF)

## References

[1] Milena E., Maurizio M. (2025). Exploring the Cardiovascular Benefits of Extra Virgin
Olive Oil: Insights into Mechanisms and Therapeutic Potential. Biomolecules.

[2] Viejo C. G., Fuentes S. (2022). Digital Detection of Olive Oil Rancidity Levels and
Aroma Profiles Using Near-Infrared Spectroscopy, a Low-Cost Electronic
Nose and Machine Learning Modelling. Chemosensors.

[3] Schwingshackl L., Hoffmann G. (2014). Monounsaturated fatty
acids, olive oil and health status:
a systematic review and meta-analysis of cohort studies. Lipids Health Dis..

[4] Latino M. E., De Devitiis B., Corallo A., Viscecchia R., Bimbo F. (2022). Consumer Acceptance
and Preference for Olive Oil Attributes—A
Review. Foods.

[5] Jimenez-Lopez C., Carpena M., Lourenço-Lopes C., Gallardo-Gomez M., Lorenzo J. M., Barba F. J., Prieto M. A., Simal-Gandara J. (2020). Bioactive
Compounds and Quality of Extra Virgin Olive Oil. Foods.

[6] Tarapoulouzi M., Agriopoulou S., Koidis A., Proestos C., Enshasy H. A. E., Varzakas T. (2022). Recent Advances
in Analytical Methods for the Detection
of Olive Oil Oxidation Status during Storage along with Chemometrics,
Authenticity and Fraud Studies. Biomolecules.

[7] Hernández M. L., Velázquez-Palmero D., Sicardo M. D., Fernández J. E., Diaz-Espejo A., Martínez-Rivas J. M. (2018). Effect of a regulated
deficit irrigation strategy in a hedgerow ‘Arbequina’
olive orchard on the mesocarp fatty acid composition and desaturase
gene expression with respect to olive oil quality. Agric. Water Manage..

[8] Gharby S., Asbbane A., Ahmed M. N., Gagour J., Hallouch O., Oubannin S., Bijla L., Goh K. W., Bouyahya A., Ibourki M. (2025). Vegetable oil oxidation: Mechanisms,
impacts on quality,
and approaches to enhance shelf life. Food chemistry:
X.

[9] Shahidi F., Zhong Y. (2015). Measurement of antioxidant
activity. J. Funct.
Foods.

[10] Karami H., Rasekh M., Mirzaee-Ghaleh E. (2020). Qualitative analysis of edible oil
oxidation using an olfactory machine. J. Food
Meas. Charact..

[11] Conte L., Bendini A., Valli E., Lucci P., Moret S., Maquet A., Lacoste F., Brereton P., García-González D. L., Moreda W., Gallina
Toschi T. (2020). Olive oil quality and authenticity:
A review of current EU legislation, standards, relevant methods of
analyses, their drawbacks and recommendations for the future. Trends Food Sci. Technol..

[12] Gertz C., Matthäus B., Willenberg I. (2020). Detection of Soft-Deodorized Olive
Oil and Refined Vegetable Oils in Virgin Olive Oil Using Near Infrared
Spectroscopy and Traditional Analytical Parameters. Eur. J. Lipid Sci. Technol..

[13] Cavanna D., Hurkova K., Džuman Z., Serani A., Serani M., Dall’Asta C., Tomaniova M., Hajslova J., Suman M. (2020). A Non-Targeted
High-Resolution Mass Spectrometry Study for Extra Virgin Olive Oil
Adulteration with Soft Refined Oils: Preliminary Findings from Two
Different Laboratories. ACS Omega.

[14] Lozano-Castellón J., López-Yerena A., Domínguez-López I., Siscart-Serra A., Fraga N., Sámano S., López-Sabater C., Lamuela-Raventós R. M., Vallverdú-Queralt A., Pérez M. (2022). Extra virgin olive oil: A comprehensive review of efforts
to ensure its authenticity, traceability, and safety. Compr. Rev. Food Sci. Food Saf..

[15] Pereira A. G., Otero P., Fraga-Corral M., Garcia-Oliveira P., Carpena M., Prieto M. A., Simal-Gandara J. (2021). State-of-the-Art
of Analytical Techniques to Determine Food Fraud in Olive Oils. Foods.

[16] Aparicio R., Rocha S. M., Delgadillo I., Morales M. T. (2000). Detection of rancid
defect in virgin olive oil by the electronic nose. J. Agric. Food Chem..

[17] Martín-Tornero E., Barea-Ramos J. D., Lozano J., Durán-Merás I., Martín-Vertedor D. (2023). E-Nose Quality Evaluation of Extra
Virgin Olive Oil Stored in Different Containers. Chemosensors.

[18] Rabehi A., Helal H., Zappa D., Comini E. (2024). Advancements and Prospects
of Electronic Nose in Various Applications: A Comprehensive Review. Appl. Sci..

[19] Karami H., Rasekh M., Mirzaee–Ghaleh E. (2020). Comparison of chemometrics
and AOCS official methods for predicting the shelf life of edible
oil. Chemom. Intell. Lab. Syst..

[20] Aghili N. S., Rasekh M., Karami H., Azizi V., Gancarz M. (2022). Detection
of fraud in sesame oil with the help of artificial intelligence combined
with chemometrics methods and chemical compounds characterization
by gas chromatography–mass spectrometry. LWT.

[21] Yang X., Pei J., He X., Wang Y., Wang L., Shen F., Li P., Fang Y. (2024). A novel method for determination of peroxide value
and acid value of extra-virgin olive oil based on fluorescence internal
filtering effect correction. Food Chem..

[22] Amelio M. (2016). The official
method for olive oil sensory evaluation: An expository revision of
certain sections of the method and a viable means for confirming the
attribute intensities. Trends Food Sci. Technol..

[23] Amelio M. (2019). Olive oil
sensory evaluation: An alternative to the robust coefficient of variation
(CVr %) for measuring panel group performance in official tasting
sessions. Trends Food Sci. Technol..

[24] Rodrigues N., Peres A. M., Baptista P., Pereira J. A. (2022). Olive Oil Sensory
Analysis as a Tool to Preserve and Valorize the Heritage of Centenarian
Olive Trees. Plants.

[25] Sipos L., Nyitrai Á., Hitka G., Friedrich L. F., Kókai Z. (2021). Sensory Panel Performance Evaluation—Comprehensive
Review of Practical Approaches. Appl. Sci..

[26] Arroyo-Cerezo A., Yang X., Jiménez-Carvelo A. M., Pellegrino M., Savino A. F., Berzaghi P. (2024). Assessment of extra
virgin olive
oil quality by miniaturized near infrared instruments in a rapid and
non-destructive procedure. Food Chem..

[27] Fernandez L., Oller-Moreno S., Fonollosa J., Garrido-Delgado R., Arce L., Martín-Gómez A., Marco S., Pardo A. (2025). Signal Preprocessing in Instrument-Based
Electronic Noses Leads to
Parsimonious Predictive Models: Application to Olive Oil Quality Control. Sensors.

[28] Karami H., Kamruzzaman M., Covington J. A., Hassouna M.é., Darvishi Y., Ueland M., Fuentes S., Gancarz M. (2024). Advanced evaluation
techniques: Gas sensor networks, machine learning, and chemometrics
for fraud detection in plant and animal products. Sens. Actuators, A.

[29] Karami H., Rasekh M., Mirzaee-Ghaleh E. (2020). Application
of the E-nose machine
system to detect adulterations in mixed edible oils using chemometrics
methods. J. Food Process. Preserv.

[30] Cano
Marchal P., Sanmartin C., Martinez S. S., Ortega J. G., Mencarelli F., Garcia J. G. (2021). Prediction of fruity aroma intensity
and defect presence in virgin olive oil using an electronic nose. Sensors.

[31] Lerma-García M., Cerretani L., Cevoli C., Simó-Alfonso E., Bendini A., Toschi T. G. (2010). Use of
electronic nose to determine
defect percentage in oils. Comparison with sensory panel results. Sens. Actuators, B.

[32] Cosio M. S., Ballabio D., Benedetti S., Gigliotti C. (2007). Evaluation
of different storage conditions of extra virgin olive oils with an
innovative recognition tool built by means of electronic nose and
electronic tongue. Food Chem..

[33] Savarese, M. ; Caporaso, N. ; Parisini, C. ; Paduano, A. ; E., de Marco ; Sacchi, R. , Application of an Electronic Nose for the Evaluation of Rancidity and Shelf life in Virgin Olive Oil, 2013.

[34] Xu L., Yu X., Liu L., Zhang R. (2016). A novel method for qualitative analysis
of edible oil oxidation using an electronic nose. Food Chem..

[35] Santonico M., Grasso S., Genova F., Zompanti A., Parente F. R., Pennazza G. (2015). Unmasking of Olive Oil Adulteration Via a Multi-Sensor
Platform. Sensors.

[36] Mariotti R., Núñez-Carmona E., Genzardi D., Pandolfi S., Sberveglieri V., Mousavi S. (2022). Volatile Olfactory Profiles of Umbrian
Extra Virgin Olive Oils and Their Discrimination through MOX Chemical
Sensors. Sensors.

[37] Modesti M., Taglieri I., Bianchi A., Tonacci A., Sansone F., Bellincontro A., Venturi F., Sanmartin C. (2021). E-Nose and
Olfactory Assessment: Teamwork or a Challenge to the Last Data? The
Case of Virgin Olive Oil Stability and Shelf Life. Appl. Sci..

[38] Casadei E., Valli E., Panni F., Donarski J., Farrús
Gubern J., Lucci P., Conte L., Lacoste F., Maquet A., Brereton P., Bendini A., Gallina
Toschi T. (2021). Emerging trends in olive oil fraud and possible countermeasures. Food Control.

[39] Valli E., Bendini A., Berardinelli A., Ragni L., Riccò B., Grossi M., Toschi T. G. (2016). Rapid and
innovative instrumental
approaches for quality and authenticity of olive oils. Eur. J. Lipid Sci. Technol..

[40] Rudnitskaya, A. Calibration Update and Drift Correction for Electronic Noses and Tongues Front. Chem. 2018; Vol. 6 10.3389/fchem.2018.00433. PMC6167416 30320065

[41] Fonollosa J., Fernández L., Gutiérrez-Gálvez A., Huerta R., Marco S. (2016). Calibration transfer and drift counteraction
in chemical sensor arrays using Direct Standardization. Sens. Actuators, B.

[42] Padilla M., Perera A., Montoliu I., Chaudry A., Persaud K., Marco S. (2010). Drift compensation of gas sensor
array data by Orthogonal Signal
Correction. Chemom. Intell. Lab. Syst..

[43] Sun F., Sun R., Yan J. (2022). Cross-Domain
Active Learning for Electronic Nose Drift
Compensation. Micromachines.

[44] Dubreuil R., Maho P., Pourtier F., Yilmaz H., Herrier C., Mouflih R., Livache T. (2022). Ammonia Detection
using a Peptide-Based
Optoelectronic Nose. Chem. Eng. Trans..

[45] He H.-J., da Silva
Ferreira M. V., Wu Q., Karami H., Kamruzzaman M. (2025). Portable and
miniature sensors in supply chain for food authentication: a review. Crit. Rev. Food Sci. Nutr..

[46] Trade Standard Applying to Olive Oils and Olive-Pomace Oils International Olive Council (IOC): Madrid, Spain; 2022.

[47] Kele M., Guiochon G. (2001). Repeatability and reproducibility
of retention data
and band profiles on reversed-phase liquid chromatography columns:
V. Results obtained with Vydac 218TP C18 columns. J. Chromatogr. A.

[48] Determination of Free Fatty Acids International Olive Council (IOC): Madrid, Spain; 2017.

[49] Determination of Peroxide Value International Olive Council (IOC): Madrid, Spain; 2017.

[50] Spectrophotometric Investigation in the Ultraviolet International Olive Council (IOC): Madrid, Spain; 2019.

[51] Singh P., Habiba U., Shafi Z., Noor A., Pandey V. K., Singh R. (2025). Understanding the Concepts
of Smart E-Nose Technology in Combination
With Machine Learning for New Era of Food Safety: An Advanced Review. Food Saf. Health.

[52] Efendi, Y. ; Wardoyo, A. Y. P. ; Naba, A. In Optimization of E-Nose Technology Using Learning Methods based on Feature Selection Algorithm, 2024 IEEE International Conference on Smart Mechatronics (ICSMech); IEEE, 2024; pp 192–197.

[53] Tiwari, A. Chapter 2 - Supervised learning: From theory to applications. In Artificial Intelligence and Machine Learning for EDGE Computing; Pandey, R. ; Khatri, S. K. ; Singh, N. k. ; Verma, P. , Eds.; Academic Press, 2022; pp 23–32.

[54] Singh, P. ; Singh, N. ; Singh, K. K. ; Singh, A. Chapter 5 - Diagnosing of disease using machine learning. In Machine Learning and the Internet of Medical Things in Healthcare; Singh, K. K. ; Elhoseny, M. ; Singh, A. ; Elngar, A. A. , Eds.; Academic Press, 2021; pp 89–111.

[55] Mohammadian N., Ziaiifar A. M., Mirzaee-Ghaleh E., Kashaninejad M., Karami H. (2024). Gas sensor technology and AI: Forecasting
lemon juice
quality dynamics during the storage period. J. Stored Prod. Res..

[56] Karami H., Khoshrou A. (2026). A novel VOC mixtures classification
methods based on
GBLinear and TabNet and informative feature selection from gas sensors
(E-Nose) data. Talanta.

[57] Pan Y., Xiao Y., Zhu M., Guo D., Ming K., Wang S., Liu X., Wang C., Xu K. (2025). Impact of
ginger juice processing on volatile compounds and sensory characteristics
of Eucommiae Cortex: A GC × GC-TOF-MS, E-nose, and machine learning
analysis. J. Pharm. Biomed. Anal..

[58] Nath, V. G. ; Bharath, S. P. ; Dsouza, A. ; Subramanian, A. Machine Learning Algorithms for Smart Gas Sensor Arrays. In Nanostructured Materials for Electronic Nose; Joshi, N. J. ; Navale, S. , Eds.; Springer Nature Singapore: Singapore, 2024; pp 185–225.

[59] Choe E., Min D. B. (2006). Mechanisms and Factors
for Edible Oil Oxidation. Compr. Rev. Food Sci.
Food Saf..

[60] Ordukaya E., Karlik B. (2017). Quality Control of Olive Oils Using Machine Learning
and Electronic Nose. J. Food Quality.

[61] Poeta E., Núñez-Carmona E., Sberveglieri V., Bernal A., Lozano J., Sánchez R. (2026). MOX Sensors
for Authenticity Assessment and Adulteration Detection in Extra Virgin
Olive Oil (EVOO). Sensors.

