# Supplementary material for: Peptide-Functionalized Silicon-Photonic E‑Nose for Monitoring Oxidation in Extra Virgin Olive Oil
Source: ACS Meas Sci Au. 2026 Mar 24;6(3):715–27. doi: 10.1021/acsmeasuresciau.6c00021 (PMC13281186; doi:10.1021/acsmeasuresciau.6c00021)
Supplement: Supplementary file 1 [file tg6c00021_si_001.pdf]

Peptide-Functionalized Silicon-Photonic E-Nose for Monitoring Oxidation in Extra Virgin Olive Oil

Hamed Karami[1,*], Antonio Pardo[2,*], Luis Fernández[1,2], Kaushal Rawal[2], Santiago Marco[1,2]

[1] Department of Signal and Information Processing for Sensing Systems, Institute for Bioengineering of Catalonia (IBEC), The Barcelona Institute of Science and Technology, Baldiri Reixac 10-12, 08028 Barcelona, Spain

[2] Department of Electronics and Biomedical Engineering, Universitat de Barcelona, Martí i Franqués 1, 08028 Barcelona, Spain

* hkarami@ibecbarcelona.eu; a.pardo@ub.edu

Text S1. Cross-Validation Strategy and Data Partitioning

To ensure robust validation and to prevent information leakage, a structured data-partitioning and cross-validation strategy was adopted. The dataset consisted of 60 independent EVOO bottles (three cultivars × two oxidation states), each measured in triplicate. Replicate measurements originating from the same oil bottle were always kept together and assigned to the same subset (training or test).

For supervised modeling, the dataset was split into a training set (60%) and an independent test set (40%), maintaining balanced representation of fresh and oxidized samples. Internal validation of the training set was performed using Venetian-blind cross-validation with 10 folds and a segment thickness of one, as implemented in the PLS Toolbox (Eigenvector Research Inc., USA). This strategy systematically excludes samples along the acquisition order and provides an unbiased estimate of predictive performance. Model hyperparameters were optimized exclusively within the training set, and the independent test set was used only for final performance evaluation.

Text S2. Latent Variable Selection and Model Parsimony in PLS-DA

The number of latent variables (LVs) in the PLS-DA model was selected based on minimization of the root mean square error of cross-validation (RMSECV), rather than on variance-explained criteria. Although two latent variables are shown in the PLS-DA score plots for visualization purposes, cross-validation indicated that a single latent variable (LV1) was sufficient to capture the relevant covariance between the sensor data (X) and class membership (Y).

This parsimonious model achieved excellent discrimination between fresh and oxidized EVOO samples, with low cross-validated and external prediction errors and near-perfect classification performance on the independent test set. The results demonstrate that inclusion of additional latent variables did not yield meaningful performance gains and would only increase model complexity without improving generalization.

Table S1. Full Confusion Matrices for PLS-DA and SVM Models (Independent Test Set)

Model	Actual \ Predicted	Fresh	Oxidized
PLS-DA	Fresh	12	0
	Oxidized	1	11
SVM	Fresh	12	0
	Oxidized	0	12

Supporting Information Availability

Supporting Information is available free of charge via the ACS Publications website.
